# Does antennal sensilla pattern of different populations of *Triatoma maculata* (Hemiptera: Reduviidae) reveal phenotypic variability?

**DOI:** 10.1186/s13071-019-3856-2

**Published:** 2019-12-23

**Authors:** Josiane Nogueira Müller, Teresa Cristina Monte Gonçalves, Alice Helena Ricardo-Silva, Amanda Coutinho Souza, Francisco Maciel Santos, Rosangela Santos, Nathalia Coelho Vargas, Catarina Macedo Lopes, Ana Laura Carbajal-de-la-Fuente

**Affiliations:** 1 Laboratório Interdisciplinar de Vigilância Entomológica em Diptera e Hemiptera, Instituto Oswaldo Cruz/ Fiocruz, Rio de Janeiro, RJ Brasil; 2 Laboratório de Doenças Parasitárias, Instituto Oswaldo Cruz/Fiocruz, Rio de Janeiro, RJ Brasil; 3 Núcleo de Entomologia, Secretaria de Saúde do Estado de Roraima, Boa Vista, Brasil; 4 Laboratorio de Eco-Epidemiología, Departamento de Ecología, Genética y Evolución, Facultad de Ciencias Exactas y Naturales, Universidad de Buenos Aires, Buenos Aires, Argentina; 5 Instituto de Ecología, Genética y Evolución de Buenos Aires (IEGEBA), CONICET-Universidad de Buenos Aires, Ciudad Autónoma de Buenos Aires, Argentina; 6 Centro Nacional de Diagnóstico e Investigación en Endemo-Epidemias (CeNDIE), Administración Nacional de Laboratorios e Institutos de Salud “Dr. Carlos Malbrán” (ANLIS), Ciudad Autónoma de Buenos Aires, Argentina

**Keywords:** Triatominae populations, Antennal phenotype, Roraima, Brazil

## Abstract

**Background:**

In Brazil, *Triatoma maculata* is only found in the State of Roraima and is a vector of *Trypanosoma cruzi*, the etiological agent of Chagas disease. It occurs in wild, peridomestic and domestic habitats, with an urban infestation in Boa Vista, the capital of this Brazilian state. The aim of this study was to assess the morphological variability of the *T. maculata* antennal phenotype in three populations of Roraima State, using the antennal sensilla pattern analyzed under optical microscopy.

**Methods:**

The number and distribution of four antennal sensilla types (bristles, thin and thick walled trichoidea, and basiconic) of three Brazilian populations of *T. maculata* from Roraima State were compared. Univariate and multivariate analyses were performed.

**Results:**

The antenna of *T. maculata* presented the four types of sensilla. According to the density and distribution of the antennal sensilla characteristics, the multivariate analyses showed that the laboratory population is morphologically structured. Urban specimens showed a pronounced phenotypic variability. The main differences were observed in the pedicel segment, and between males and females.

**Conclusions:**

We determined the antennal phenotype in three Roraima populations of *T. maculata*. These results support the idea that the patterns of antennal sensilla are sensitive markers for distinct populations in the Triatominae. The infestations of *T. maculata* in different habitats reinforces the ability of this vector to become adapted to a variety of environments, which, could have eco-epidemiological implications for the *T. cruzi* transmission that are still not well understood.

## Background

Chagas disease, caused by *Trypanosoma cruzi* (Chagas, 1909), is mostly transmitted by blood-sucking bugs of the subfamily Triatominae. *Triatoma maculata* (Erichson, 1848), a vector of *T. cruzi*, is distributed in the northern regions of South America and in Brazil, where it is only found in the State of Roraima [1]. It occurs in wild habitats, in palms of the genus *Attalea* and is associated with opossums (*Didelphis marsupialis*), birds and bats [2]. In peridomestic environments, it is associated with chicken coops and pigeon nests [2]. In the State of Roraima, a domiciliary infestation of *T. maculata* was found [3] and recently, an infestation was recorded in an urban area of a residential neighbourhood in the city of Boa Vista [4].

The insects have flexibility in the expression of characters, contributing to adaptability to various environments. This flexibility is called phenotypic variability and is considered essential for understanding the development and maintenance of morphological variation [5]. In triatomines, the phenotype may vary because of the ability to feed on different hosts, to adapt to the environments where it developed, and to vary the host-vector contact rates, among other factors [2]. Consequently, phenotypic variation is an adaptive response, which can vary physiological processes in response to environmental pressures [6].

The triatomines perceive sensorial stimuli from the environment by receptors located mainly on the antennae denominated sensilla; these are classified as mechanoreceptors and chemoreceptors [7]. Among the chemoreceptive sensilla are those that detect chemical components related to food sources, recognize sexual partners, and locate preferred habitats [7]. The antennal phenotype consists of the type and number of sensilla distributed on the antenna and is considered an indicator of the adaptation of the triatomines to ecotopes of different complexities and stabilities [6]. It provides an efficient and low-cost response to determine the morphological variability among genera, species and populations [7–9].

In Brazil, *T. maculata* is considered one of the species of epidemiological importance [10]. As part of an interdisciplinary study on the eco-epidemiological aspects conducted in the area, an integral project involving research, education, and health services was conducted. The abundance and infestation of domestic and peridomestic sites by *T. maculata* were determined, as well as their infection with *T. cruzi* and their feeding sources [3]. Simultaneously, an urban infestation was detected in Boa Vista [4]. In this context, we studied the morphological variability of *T. maculata* antennal phenotype. We included peridomestic populations of Amajari, Bonfim and a population with 18 generations maintained in the laboratory (all from Roraima State). Sexual dimorphism was also explored.

## Methods

Three Brazilian populations of *T. maculata* from Roraima State were compared: from Amajari (03°39′07″N, 61°22′15″W); Bonfim (03°21′36″N, 59°49′58″W); and a laboratory colony from Uiramutã (04°35′45″N, 60°10′4″W). Triatomines were collected during 2014–2015 by active search. The field adults were collected in chicken coops, except for the laboratory colony. This colony was originated from adult specimens (*n* = 38) feeding on mice (License P0100-01 CEUA-FIOCRUZ) and is maintained in the insectary of the Laboratório Interdisciplinar de Vigilância Entomológica em Diptera e Hemiptera, Instituto Oswaldo Cruz, FIOCRUZ, Brazil. The insects studied were from a colony of 18th generation. A total of 15 males and 15 females from three populations of *T. maculata* were included in this study and were identified following a dichotomous key traditionally used for this purpose [11].

One right antenna per individual was removed using fine forceps, stored in 70% ethanol according to previous protocols [8]. Sensilla identification and counting were made on the ventral side of the three distal segments of the antenna [pedicel (P); flagellum first segment (F1); and flagellum second segment (F2)] using optical microscopy (Leica, DMLS, Wetzlar, Germany) (400×) and a drawing chamber (ISH 1000, Tucsen, Australia). Sensilla were classified as follows: bristles (BR); thin-walled trichoids (TH); thick-walled trichoids (TK); and basiconic (BA) [8]. Means and standard deviations were calculated for each type of sensilla in each of the antennal segments.

Levene’s test was used to check the homogeneity of variances. Variables were analyzed using ANOVA and mean values were contrasted using Tukey’s *post-hoc* test. Variables with significant differences were used for a discriminant analysis. Mahalanobis distances were calculated as the distance between group centroids generated by the discriminant functions. Their statistical significance was calculated through permutation tests (1000 runs each) and was corrected by means of the Bonferroni method. A cross-check classification was used to validate the classification of the individuals in the discriminant analysis. A few adult specimens (*n* = 3) from Boa Vista city (02°49′12″N, 60°40′19″W) collected at domiciles were used in this study. Because of this low number, it was not possible to incorporate them into the ANOVA. However, it was possible to include them one by one in the discriminant analysis as ‘unknown specimens’ [12]. This allowed for the determination of the similarity of each individual to the reference population. The ANOVA was carried out using JMP v. 6.0.0 (SAS Institute Inc., 2005) and discriminant analysis was performed using the CLIC v. 98 package (http://xyom-clic.eu/).

## Results

The antenna of *T. maculata* presented four types of sensilla distributed on three segments (Table 1). All variables showed variance homoscedasticity except for TH pedicel. Significant differences between the Amajari and laboratory populations were detected in the number of F2-TK sensilla (Tukeyʼs *post-hoc* tests, all *P *< 0.05). There were also significant differences for the number of F1-BR sensilla of the Amajari populations and P-BR sensilla of the laboratory population (data not shown). ANOVA test for sensilla numbers revealed significant differences between sexes (*F*_(1, 28)_ = 9.71, *P* = 0.0042). Tukey’s *post-hoc* test showed a sexual dimorphism for Amajari, Bonfim and laboratory populations mainly for sensilla on the pedicel segment (all *P *< 0.05) (Table 1).

**Table 1:** Mean number of sensilla (standard deviation) on each antennal segment of *Triatoma maculata* populations from Roraima State, Brazil

Locality	*n*	sex	Pedicel				Flagellum 1				Flagellum 2			
			BR	TH	TK	BA	BR	TH	TK	BA	BR	TH	TK	BA
Amajari	5	F	103.6 (9.2)	248.2 (35.9)^a^	45.6 (12.6)^a^	22.8 (7.3)^a^	12.4 (0.9)	193.0 (17.1)	106.4 (19.0)	47.8 (9.9)	7.6 (1.5)	118.8 (18.2)	47.4 (11.8)	40.0 (3.6)
	5	M	91.4 (10.4)	496.0 (65.2)^b^	11.8 (7.2)^b^	11.4 (2.5)^b^	12.2 (1.8)	210.8 (20.0)	107.8 (12.8)	31.2 (9.9)	7.4 (1.5)	121.8 (33.2)	46.0 (21.7)	28.2 (14.8)
Bonfim	5	F	105.6 (8.6)	275.4 (32.1)^a^	55.6 (29.1)^a^	18.8 (6.3)	14.6 (2.2)	188.2 (20.0)	122.6 (12.3)	31.2 (11.6)	9.6 (0.9)	115.4 (22.5)	69.2 (20.4)	35.0 (11.0)
	5	M	91.2 (5.1)	448.6 (61.1)^b^	6.6 (4.7)^b^	9.8 (2.2)	14.4 (1.5)	219.6 (28.7)	86.2 (12.8)	35.8 (7.0)	9.0 (1.7)	133.4 (13.4)	54.4 (6.0)	28.0 (7.3)
Laboratory	5	F	119.2 (10.0)	218.6 (23.4)	44.6 (16.9)^a^	15.8 (5.9)	14.6 (0.5)	185.2 (22.8)	116.0 (32.3)	48.4 (13.7)^a^	7.8 (3.3)	93.6 (38.6)	65.4 (27.4)	32.6 (11.8)
	5	M	107.2 (9.6)	311.4 (53.3)	13.8 (9.1)^b^	8.4 (4.3)	16.0 (2.3)	195.2 (19.2)	108.8 (24.6)	28.0 (3.5)^b^	8.8 (1.6)	103.0 (13.1)	85.8 (13.7)	36.0 (2.4)

^a, b^ Significant differences between the sexes of each population, Tukey’s *post-hoc* test (*P *< 0.05)

*Abbreviations*: *n*, number of antennae examined; F, females; M, males

*Note*: Sensilla types: BR, bristles; TH, thin-walled trichoids; TK, thick-walled trichoids; BA, basiconic

**Fig. 1:**
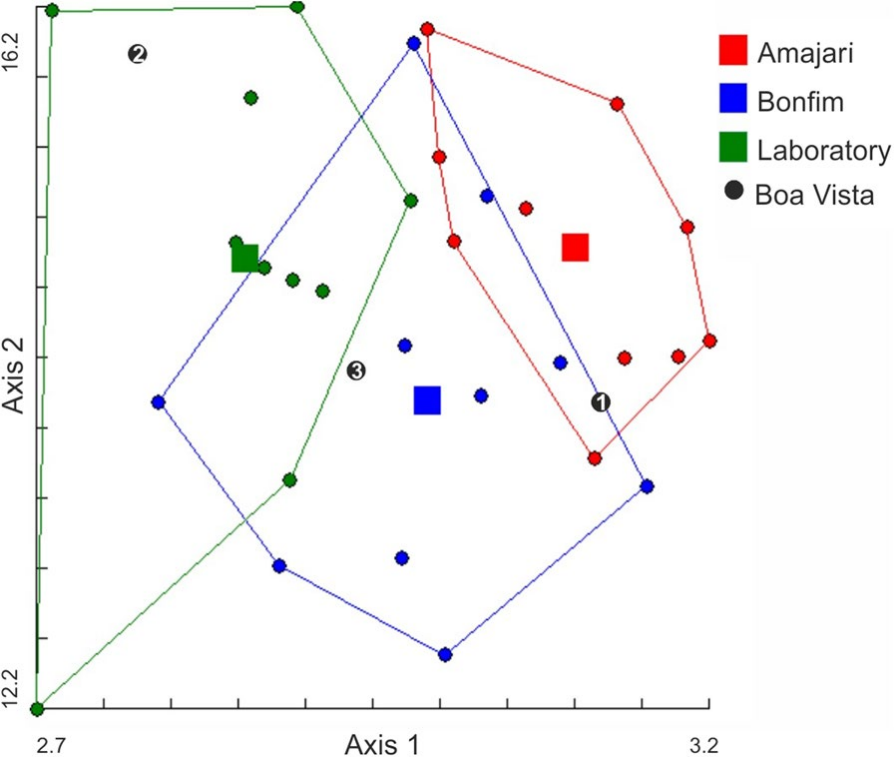
A factorial map based on significant variables of the antennal phenotype of populations of *Triatoma maculata*, Roraima State, Brazil. The lines connect the most external individuals (circles) of each population. Squares represent centroids for each population. The Mahalanobis distances showed that the laboratory population was significantly different compared with the Amajari population (*P *< 0.01) and the Bonfim population (*P *< 0.01). Urban triatomines from Boa Vista (black circles 1–3) were introduced as ‘unknown specimens’ [12]

The multivariate analysis performed using three significant variables (number of P-BR, F1-BR and F2-TK sensilla) showed that the first two discriminant factors accounted for 89% and 11% of the total variation, respectively. The factorial distribution map of the individuals in the first plane of the discriminant analysis and based on the Mahalanobis distances showed low discrimination between groups in this space (Fig. 1). The Mahalanobis distances showed that the laboratory population was significantly different from the populations of Amajari (Mahalanobis distance = 2.72; *P *< 0.01) and Bonfim (Mahalanobis distance = 1.71; *P *< 0.01). The cross-checked classification (1000 permutations) revealed that 80% of the laboratory specimens were correctly classified. However, for the individuals of the Amajari and Bonfim populations, the classification values were 60% and 40%, respectively. Because of the low number of individuals from Boa Vista (*n* = 3), they were included one by one in the discriminant analysis as ‘unknown specimens’. The results showed that they were similar to each one of three studied populations, suggesting an antennal phenotypic heterogeneity (Fig. 1).

## Discussion

The multivariate analysis showed that the laboratory population is morphologically structured and the specimens of the urban population showing phenotypic variability. Based on the univariate analysis, the main difference was found in the pedicel with the number of thick walled trichoids (TK) on this segment, being significantly different between males and females of the three populations. Sexual dimorphism in the antennal phenotype of triatomines has been reported in other Brazilian species, such as *Triatoma sordida* and *Triatoma pseudomaculata* [9] and *T. maculata* [8].

Diversity in the type and number of receptors in the pedicel was observed in four species of the genus *Triatoma*, which could be related to the characteristics of the habitat where each species evolved [9]. The pedicel of *T. maculata* populations of Amajari and Bonfim, collected from the peridomicile (chicken coop) environment, is complex, they show a higher density of sensilla than in sylvatic *T. maculata* [8]. These results suggest that species that develop in multiple habitats and are not very stable (e.g. *T. sordida*, *T. pseudomaculata*), present more types and a greater number of sensilla in the pedicel [7]. Moreover, *T. maculata* individuals raised under stable conditions of temperature, humidity, and frequent feeding in the laboratory, had a pedicel with fewer sensilla. Considering the laboratory as a new habitat for triatomines, many authors showed morphological changes associated with different rearing conditions [13] also observed in the present study in the laboratory population. In this sense, the antennal sensilla of triatomines showed a degree of morphological variability among populations that seemed to be associated with adaptations based on the sensorial requirements of different habitats [7]. Thus, it would seem logical to expect that a population developed in a laboratory habitat, which undoubtedly differs from its natural habitat, presents changes in the antennal phenotype ([13, 14]; the present study).

Our study presents some limitations, such as a low number of individuals, allowing only simple descriptive analysis. In addition, it was not possible to include a population of *T. maculata* collected in the wild, which would have allowed other comparisons in relationship to ecotopes. However, similar results were reported for the antennal phenotype of the pedicel of individuals collected in palm trees from wild areas of the state of Roraima, Brazil [8]. Microhabitats with a stable temperature and humidity, such as the base of the palm leaf where the triatomines grow, as well as stable environments with temperature, humidity, and feeding under controlled laboratory conditions, could reflect this similarity. This not only supports the idea of morphological plasticity but also suggests caution in the use of long-term laboratory material for morphological studies [14]. The individuals from the urban area of Boa Vista, which were placed in the discriminant analysis as ‘unknown’, showed phenotypic similarities with the laboratory population, the Amajari and Bonfim populations. Because this was an exploratory analysis with a low number of individuals, it was not possible to interpret the results without speculation. However, the occurrence of *T. maculata* in different habitats may suggest the ability of this vector to adapt to a variety of environments, which could have eco-epidemiological implications that are still not well known [4, 15–17]. In agreement with Noireau et al. [18], although anthropogenic environmental changes and successive damage to the habitats of triatomines could promote dispersal and favour the domiciliation process, the basic mechanisms of adaptation of these insects to artificial ecotopes remain poorly understood.

## Conclusions

We determined the antennal phenotype of three Roraima populations of *T. maculata*. The laboratory population was morphologically structured in relation to the density and distribution of the antennal sensilla. The urban individuals from Boa Vista showed a pronounced phenotypic variability. The main differences were in the pedicel and between males and females of the three populations. These results support the idea that the patterns of antennal sensilla are sensitive markers for distinct populations in the Triatominae.

## Data Availability

The datasets used and/or analyzed during the present study available from the corresponding author upon reasonable request.

## Abbreviations

P: pedicel
F1: flagellum first segment
F2: flagellum second segment
BR: bristles
TH: thin-walled trichoids
TK: thick-walled trichoids
BA: basiconic
